# Alex Langmuir and CDC

**DOI:** 10.3201/eid1210.060826

**Published:** 2006-10

**Authors:** Warren Winkelstein, Arthur L. Reingold

**Affiliations:** * University of California School of Public Health, Berkeley, California, USA

**Keywords:** CDC, Alex Langmuir, Joe Mountin

**To the Editor:** We were surprised and disappointed by the brevity of your article commemorating the 60th anniversary of the establishment of the Communicable Disease Center (CDC) (*1*). We realize that the accomplishments of the center and its derivative agencies are vast and that to give them full recognition would require far more space in Emerging Infectious Diseases than might be feasible. Nevertheless, your article that appropriately identified Joe Mountin as the administrative "father" of the center omitted any mention of Alex Langmuir, arguably the most influential of the infectious disease leaders over the years. Langmuir's creation and direction of the Epidemic Intelligence Service epitomized CDC's role in infectious diseases. His legacy deserves recognition in any chronicle of CDC, no matter how short.
