# Supplementary material for: Alex Langmuir and CDC
Source: Emerg Infect Dis. 2006 Oct;12(10):1619. doi: 10.3201/eid1210.060826 (PMC3293460; doi:10.3201/eid1210.060826)
Supplement: Related texts

# Reply

**DOI:** 10.3201/eid1210.060851

**Authors:** Tanja Popovic

**Affiliations:** * Centers for Disease Control and Prevention, Atlanta, Georgia, USA

**In response**: We thank Drs. Winkelstein and Reingold (*1*) for their comment regarding our article on the 60 years of CDC's progress in the area of infectious diseases. We certainly agree that Dr. Alexander Langmuir has made enormous contributions to this area, which we fully respect, We further agree that a more comprehensive approach would have required far more space than allotted for these types of commentaries. Allow us to emphasize that the omission of Dr. Langmuir and many other outstanding colleagues was not an oversight but an effort on our part to abbreviate an exceptionally long list of these deserving persons. We are looking forward to opportunities to provide a more comprehensive overview in which many of them will be appropriately recognized.

## References

[1] Winkelstein W Jr, Reingold AL. Alex Langmuir and CDC [letter]. Emerg Infect Dis. 2006;12:1619.1717659410.3201/eid1210.060826PMC3293460
